# Molecular Epidemiological Investigation of a Nosocomial Cluster of *C. auris*: Evidence of Recent Emergence in Italy and Ease of Transmission during the COVID-19 Pandemic

**DOI:** 10.3390/jof7020140

**Published:** 2021-02-15

**Authors:** Vincenzo Di Pilato, Giulia Codda, Lorenzo Ball, Daniele Roberto Giacobbe, Edward Willison, Malgorzata Mikulska, Laura Magnasco, Francesca Crea, Antonio Vena, Paolo Pelosi, Matteo Bassetti, Anna Marchese

**Affiliations:** 1 Department of Surgical Sciences and Integrated Diagnostics (DISC), University of Genoa, 16132 Genoa, Italy; giulia.codda@gmail.com (G.C.); lorenzo.ball@edu.unige.it (L.B.); paolo.pelosi@unige.it (P.P.); anna.marchese@unige.it (A.M.); 2 Anesthesia and Intensive Care, San Martino Policlinico Hospital—IRCCS for Oncology and Neuroscience, 16132 Genoa, Italy; 3 Infectious Diseases Unit, San Martino Policlinico Hospital—IRCCS for Oncology and Neuroscience, 16132 Genoa, Italy; daniele.roberto.giacobbe@gmail.com (D.R.G.); m.mikulska@unige.it (M.M.); lmagnasco90@gmail.com (L.M.); anton.vena@gmail.com (A.V.); matteo.bassetti@hsanmartino.it (M.B.); 4 Department of Health Sciences (DISSAL), University of Genoa, 16132 Genoa, Italy; 5 Clinical Microbiology Unit, San Martino Policlinico Hospital—IRCCS for Oncology and Neuroscience, 16132 Genoa, Italy; willisonedward@gmail.com (E.W.); francesca.crea@hsanmartino.it (F.C.)

**Keywords:** mycotic infections, antifungal resistance, genomic epidemiology, COVID-19, epidemic, emerging infections

## Abstract

*Candida auris* is an emerging MDR pathogen raising major concerns worldwide. In Italy, it was first and only identified in July 2019 in our hospital (San Martino Hospital, Genoa), where infection or colonization cases have been increasingly recognized during the following months. To gain insights into the introduction, transmission dynamics, and resistance traits of this fungal pathogen, consecutive *C. auris* isolates collected from July 2019 to May 2020 (*n* = 10) were subjected to whole-genome sequencing (WGS) and antifungal susceptibility testing (AST); patients’ clinical and trace data were also collected. WGS resolved all isolates within the genetic clade I (South Asian) and showed that all but one were part of a cluster likely stemming from the index case. Phylogenetic molecular clock analyses predicted a recent introduction (May 2019) in the hospital setting and suggested that most transmissions were associated with a ward converted to a COVID-19-dedicated ICU during the pandemic. All isolates were resistant to amphotericin B, voriconazole, and fluconazole at high-level, owing to mutations in *ERG11*(K143R) and *TACB1*(A640V). Present data demonstrated that the introduction of MDR *C. auris* in Italy was a recent event and suggested that its spread could have been facilitated by the COVID-19 pandemic. Continued efforts to implement stringent infection prevention and control strategies are warranted to limit the spread of this emerging pathogen within the healthcare system.

## 1. Introduction

*Candida* species are one of the most common fungal pathogens causing invasive infections at a global scale [1]. Over past years, *Candida auris* has emerged as a fungal pathogen of major concern, frequently showing a multidrug-resistant (MDR) phenotype and being recognized as an important cause of nosocomial outbreaks of invasive infections worldwide [2]. The global dissemination of this pathogen has been initially attributed to the simultaneous emergence of four major phylogenetically distinct lineages, showing a specific geographical distribution and including: South Asian (I), East Asian (II), African (III), and South American (IV) clades [3]. A potential fifth clade of Iranian origin has been also described [4]. More recently, however, some evidence for an increased phylogeographic mixing has been provided [5], since isolates from countries of most global regions appeared interspersed in phylogenies (i.e., primarily in clade I and III), a phenomenon likely contributed by global travels of persons with prior healthcare exposures to *C. auris*.

Most of reported infections occurred in immunocompromised and/or critically ill patients with severe underlying diseases and were associated with high mortality rates, as the frequent resistance phenotype to multiple antifungal drugs accounts for high rates of clinical treatment failures [6]. Although factors predisposing to *Candida* infections appear similar, regardless of the species, it has been recently shown that longer stay in ICU, respiratory disease, vascular surgery, presence of CVC, urinary catheter, surgery within past 30 days, and prolonged exposure to antifungal drugs are the most common risk factors for acquisition of *C. auris* [6,7,8]. Moreover, the ability to persist in the hospital environment and to colonize multiple human anatomical sites poses major risks for the intrahospital transmission of this fungal pathogen and could represent an additional risk factor for development of invasive candidiasis [9,10]. For these reasons, the impact that *C. auris* could have in the clinical setting is an ever-growing concern.

Since 2015, either sporadic cases or nosocomial outbreaks have been documented in several Europe countries, primarily in Spain and the UK, where intra- and inter-facility transmissions have been described [11]. In Italy, the index case of *C. auris* was identified in 2019 in our hospital (San Martino Hospital, Genoa), which is a large teaching facility located in the northern area of the country [12]; subsequently, no more reports have been documented in our country.

In this study, we report on novel *C. auris* cases recently recognized in our hospital after the initial description of the index case and being likely associated with a nosocomial cluster. A molecular epidemiological investigation was carried out to provide insights into the introduction, the putative transmission dynamics, and the resistance traits of this fungal pathogen.

## 2. Materials and Methods

### 2.1. *Candida auris* Isolates

A total of 10 nonduplicate clinical isolates were collected from individual patients admitted to the San Martino Hospital (Genoa, Italy) during the period spanning July 2019–May 2020 and diagnosed with invasive infections (blood, *n* = 5) or colonization (broncho-alveolar lavage, urine, *n* = 5) by *C. auris*. All isolates were cultivated using Liofilchem Chromatic *Candida* (Liofilchem, Roseto degli Abruzzi, Italy) and grown for 48 h at 30 °C; frozen stocks were subsequently prepared with 15% sterile glycerol and stored at −80 °C. The species-level identification was carried out by matrix-assisted laser desorption ionization-time of flight mass spectrometry (MALDI-TOF MS) (Vitek MS; bioMérieux, Marcy-l’Etoile, France) using the VITEK MS software v4,0 and confirmed by PCR amplification of the species-specific GPI protein-encoding genes, as previously described [12,13].

### 2.2. Antifungal Susceptibility Testing (AFST)Share and Cite

Antifungal susceptibility testing of nine antifungal agents (i.e., amphotericin B, flucytosine, fluconazole, itraconazole, voriconazole, posaconazole, caspofungin, anidulafungin, and micafungin) was performed according to the CLSI M27-A3 guidelines [14], using commercial broth microdilution plates (Sensititre YeastOne, Thermo Scientific, Waltham, MA, USA) and the manufacturer’s instructions. As *C. auris*-specific susceptibility breakpoints have not yet been established, tentative breakpoints proposed by the CDC (https://www.cdc.gov/fungal/candida-auris/c-auris-antifungal.html, accessed 10 January 2021) or previously adopted by other studies were used [15]. In brief, resistance breakpoints were defined as follows: micafungin at ≥4 μg/mL, caspofungin at ≥2 μg/mL, anidulafungin at ≥4 μg/mL, amphotericin B at ≥2 µg/mL, 5-fluorocytosine at ≥ 128 μg/mL, fluconazole at ≥32 μg/mL, and voriconazole at ≥2 µg/mL.

### 2.3. Whole-Genome Sequencing (WGS)

Total genomic DNAs was extracted from broth cultures using the Qiagen DNeasy PowerLyzer PowerSoil Kit (Qiagen, Hilden, Germany). Shotgun libraries were prepared from purified DNAs and sequenced with a 2 × 150 bp paired-end approach using the Illumina HiSeq platform (Illumina, Inc., San Diego, CA, USA) at Novogene Co., Ltd. (Cambridge, United Kingdom). The *C. auris* index strain (FG_GE01) was further sequenced using the MinION sequencer (Oxford Nanopore Technologies, Oxford, UK) to generate a complete, cluster-specific, reference genome using Flye v2.8 [16], Medaka v1.0.3 (https://github.com/nanoporetech/medaka, accessed on 10 September 2020) and Pilon v1.22 [17], as previously described [18].

### 2.4. Phylogenomic Reconstruction and Comparative Analyses

Clonal relatedness was evaluated by core-genome single-nucleotide polymorphisms (SNPs) using BWA v0.7.17 to align the Illumina reads against the *C. auris* FG_GE01 reference genome [19], and NUCmer v3.1 and FreeBayes v1.1.0 to identify repetitive regions and call SNPs, respectively [20,21]. Maximum-likelihood phylogenetic trees were constructed on concatenated core-genome SNPs using IQ-TREE v1.6.12, using a general time reversible (GTR) nucleotide substitution model [22]. The tree topology was estimated using TempEst v1.5.159 and calibrated with sampling times [23]; a root-to-tip regression was then calculated, and the root of the tree was selected to maximize R2, as previously described [24]. Estimates of times of the most recent common ancestor (tMRCA) were performed in a Bayesian framework using a Markov chain Monte Carlo (MCMC) method implemented in BEAST v.1.10.4 [25], as previously described [5]. Raw reads of *C. auris* isolates used in comparative analyses were retrieved from the NCBI SRA database (https://www.ncbi.nlm.nih.gov/sra/?term=candida+auris, accessed on 9 December 2020), through the RunSelecter tool; a total of 871 samples were selected using the following inclusion criteria: assay type: WGS; organism: *Candida auirs*; host: homo sapiens; Instrument: Illumina MiSeq, iSeq, HiSeq, NextSeq, NovaSeq; sequenced megabases: > 200; available information about the geographic location (Table S1). The genome sequence of the *C. auris* strain B8441 (Acc. no: PEKT00000000.2) was used as reference to verify the presence of mutation possibly involved in fluconazole and amphotericin B resistance. Sequence comparisons were performed by BLAST (https://blast.ncbi.nlm.nih.gov/blast/, accessed on 4 February2021).

## 3. Results

### 3.1. Case Series Description

**Figure 1:**
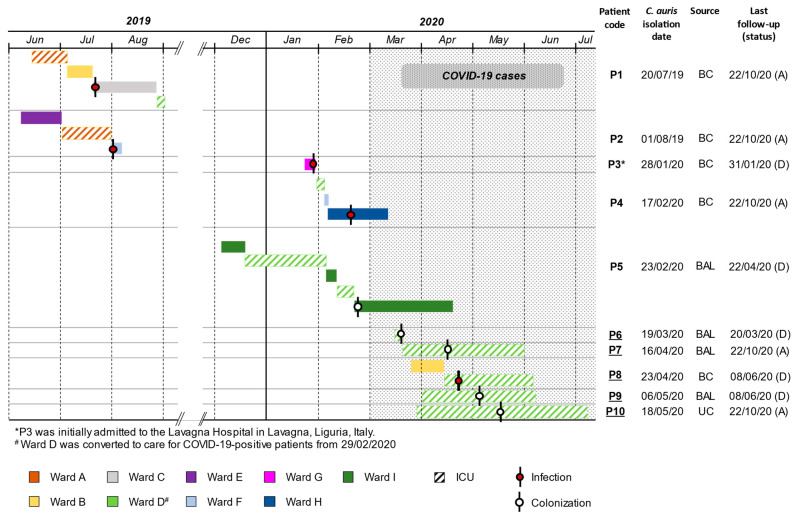
Overview of patient traces within different HSM hospital wards in the period from June 2019 to July 2020. Isolation of *C. auris* is indicated by black bars, marked with a red- or white-filled circle in case of infection or colonization events, respectively. Patients diagnosed with COVID-19 are underlined. Abbreviations: BC, blood culture; BAL, broncho-alveolar lavage; UC, urine culture; A, alive; D, dead.

The index case of *C. auris* has been reported in Italy in mid-July 2019 [11], from an intensive care unit (ICU) patient (P1) admitted to the San Martino Hospital (HSM) where, in early August 2019, a second patient (P2) was diagnosed with a bloodstream infection (BSI) by *C. auris*. After a few months, in late January 2020, an additional case was recorded in a patient (P3) initially diagnosed with a *C. auris* BSI (isolation date: 15/01/20) at the Lavagna Hospital (Lavagna, Italy), another healthcare facility located in the same regional area, and then transferred to the HSM for further treatment. Subsequently, following the emergence of the COVID-19 pandemic, episodes of BSI (P4, P8) and cases categorized as colonization (P5, P6, P7, P9, P10) by *C. auris* have been increasingly reported in patients admitted to the HSM from February to May 2020 (Figure 1). Overall, a total of 10 consecutive isolates of *C. auris*, accounting for 10 cases including the index patient, were collected and then subjected to further molecular and phenotypic investigations.

In addition, the clinical characteristics of COVID-19 patients diagnosed with infection or colonization by *C. auris* in our hospital were examined, and results were presented in a dedicated report [26].

### 3.2. Inference of Putative Origin and Emergence of *C. auris* Isolates in Italy

**Figure 2:**
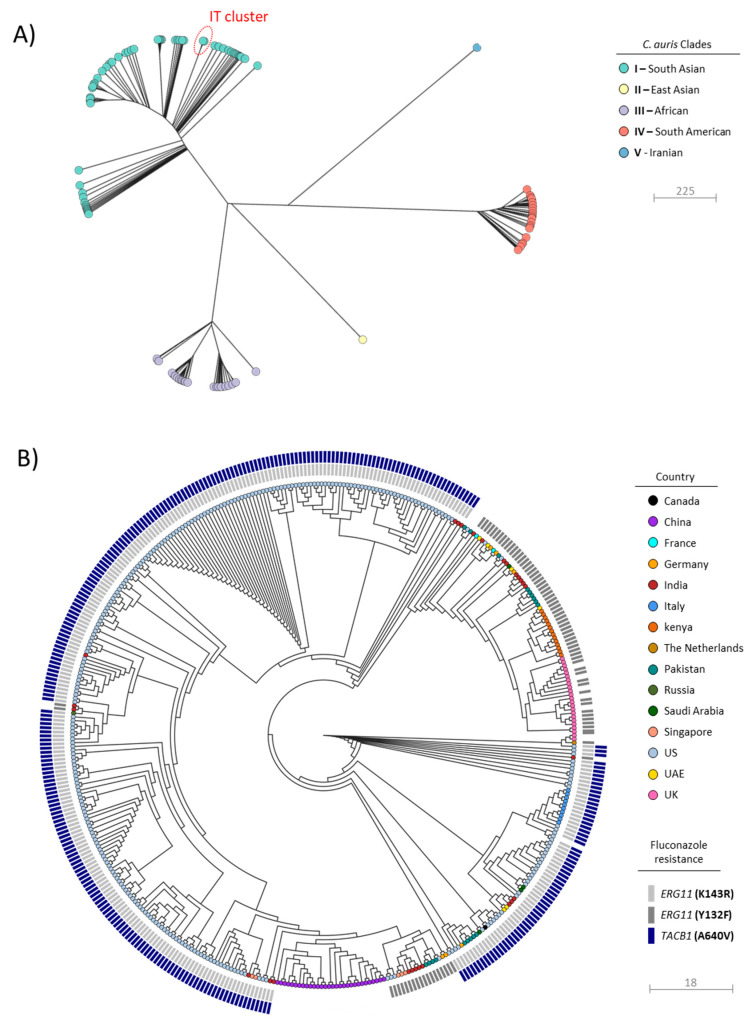
Global phylogenetic analysis of *C. auris*, including genomes retrieved in this study (*n* = 10) and representative genomes from different countries and clades (*n* = 871) (Table S1). (**A**) Maximum-likelihood phylogenetic tree of *C. auris* whole-genome sequences (*n* = 881) clustering into four major clades. The colors of the isolate tips represent the different clades. The branch comprising the *C. auris* isolates from Italy is circled in red. (**B**) Maximum-likelihood phylogenetic tree of *C. auris* belonging to the clade I (*n* = 426). The colors of the isolate tips represent the country of isolation. Details about the genetic mutations (*ERG11*, *TAC1B*) associated with high-level resistance to fluconazole were also provided.

In order to rule out the simultaneous emergence of multiple *C. auris* lineages and to further infer the origin of collected isolates, high-resolution genotyping through WGS was performed. According to sequencing results, the species-level identification as *C. auris* was confirmed for all isolates, and a complete reference genome (i.e., including seven linear chromosomes and one circular mitochondrial DNA molecule) to be used in downstream analyses was generated for *C. auris* FG_GE01 (Acc. Nos CP061160-CP061167), representing the Italian index case. A global phylogenetic analysis, including a collection of 871 genomes from 25 countries on six continents (Table S1), was then performed and results showed that all isolates from HSM were resolved within the South Asian clade (I) (Figure 2A). An additional phylodynamic analysis restricted to members of this clade, which included 416 isolates from Canada, China, France, Germany, India, Kenya, the Netherlands, Pakistan, Russia, Saudi Arabia, Singapore, the US, United Arab Emirates, and the UK, revealed that isolates from Italy segregated in a monophyletic clade (SNPs median\mean: 20) together with five isolates from the US (mainly from New Jersey). Previous epidemiological investigations including the latter isolates revealed their introduction route in the US was unknown, since no links to healthcare exposures abroad were documented [27].

Interestingly, following a root-to-tip regression analysis, a correlation between sampling dates and the genetic distances among sequence isolates in this study was observed (correlation coefficient: 0.89; R2 = 0.80), indicating that the sequence dataset had a sufficient phylogenetic temporal signal to perform further molecular clock analyses. A more accurate estimate of the time to the most recent common ancestor of sequenced isolates (i.e., the time since they last shared a common ancestor) corresponded to 27 May 2019 (95% highest posterior density interval: 27 December 2018–16 July 2019), less than two months prior to the identification of the *C. auris* index case in Italy, indicating a relatively recent introduction event.

### 3.3. Investigation of Clonal Relatedness and Putative Transmission Patterns of *C. auris* from HSM

**Figure 3:**
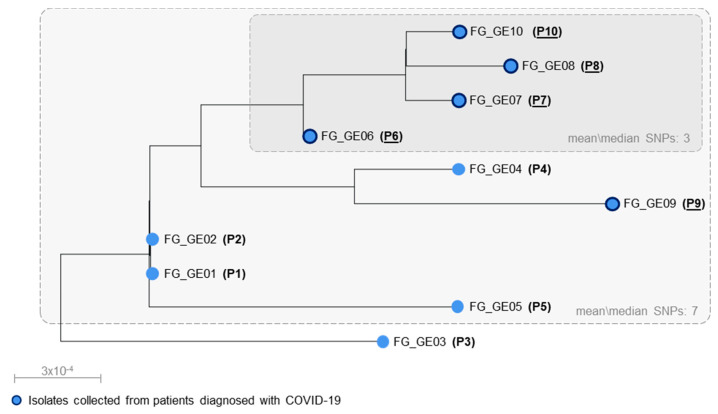
Phylogenetic analysis of *C. auris* isolates sequenced in this study. The phylogenetic tree topology was calibrated with sampling times and estimated using TempEst [23]. For each tip, the *C. auris* strain name and the patient’s code are provided. Patients diagnosed with COVID-19 are underlined, and the corresponding isolates’ tips are marked with dark blue tick borders.

A phylogenetic analysis restricted to the *C. auris* isolates from HSM suggested that 9 out of 10 of them were most likely associated with a nosocomial cluster (SNPs mean\median: 7) (Figure 3), consistent with previous evidence about the genetic diversity observed among pairs of epidemiologically linked cases of *C. auris* (median SNPs difference: 7) [26]. The remaining isolate, obtained from P3, most likely represented a member of the same *C. auris* lineage accounting for a separate introduction (SNPs against other isolates: mean, 12; median, 14), consistently with its original identification in a different hospital.

A combined analysis of clonal and patient trace data suggested that the intrahospital spread of *C. auris* likely stemmed from the index patient, occurred under different time frames, and involved two specific wards of HSM (Figure 1). The ward A was possibly related to the initial emergence and transmission of *C. auris* between the first two patients, P1 and P2, who were infected by genetically identical isolates (0 SNPs) and had a concomitant stay in this ICU. The ward D was associated with a putative horizontal transmission between the other seven patients, of which five (P6 to P10) were admitted as COVID-19 patients during the early phase of the pandemic (Figure 1). In this case, however, more complex dynamics were possibly involved, since transmissions supported by WGS were not fully explained by patients’ overlap. Independent transmissions from P1 to P4, P5, P6, and P9 (SNPs mean\median: 8) were deemed and likely involved intermediate patients/operators or contaminated environmental surfaces in ward D, where *C. auris* could have been primarily introduced by the index patient during its stay (Figure 1 and Figure 3). Former investigations, indeed, showed that *C. auris* was successfully cultivated from axillary and ear swabs obtained from P1 [12], who could have shed large amounts of this organism from her skin, thus leading to contamination of surrounding environments. Furthermore, P4, P5, P6, and P9 occupied adjacent beds in a defined area of ward D, being in the proximity to the bed initially occupied by P1. A transmission chain, on the other hand, was predicted among the remaining patients, likely going from P6 through P7, P8 and P10 as suggested by the high genetic relatedness of corresponding *C. auris* isolates (SNPs mean\median: 3) and by their overlap in ward D (Figure 1 and Figure 3).

### 3.4. Antifungal Susceptibility Profiles

Results from AFST revealed that all tested isolates were resistant to fluconazole, voriconazole, and amphotericin B. Conversely, lower MIC values were observed for the other tested antifungal drugs (Table 1). Inspection of the *ERG2*, *ERG3*, *ERG5*, and *ERG6* coding sequence in *C. auris* genomes from Italy revealed no mutations previously associated with amphotericin B resistance [28,29,30]. Conversely, the presence of the same amino acid substitution *ERG11*(K143R), being previously shown to reduce azole susceptibility [3], has been detected in all isolates. A genetic alteration recently associated with a high-level resistance to fluconazole, due to a transcriptional upregulation of ABC-type efflux pumps, was additionally identified in all sequenced isolates in *TAC1B*(A640V) [31]. Interestingly, following a large-scale analysis of WGS data aimed at evaluating the distribution and frequency of the *TAC1B*(A640V) substitution over 400 *C. auris* isolates belonging to the South Asian clade, a strong association with the *ERG11*(K143R) variant was observed at a global level (Figure 2B), corroborating a significant contribution of both alterations to clinical fluconazole resistance. Nevertheless, multiple factors seem to contribute to azole resistance in *C. auris*, since mutation in *ERG11* have been also identified in azole-susceptible isolates [32]; further investigations are therefore warranted to elucidate the genetic bases of this resistance mechanism.

## 4. Discussion

The global spread of *C. auris* poses a major threat to healthcare system. Hence, tracking the dissemination of this emerging pathogen and understanding its introduction and transmission dynamics, as well as its resistance features, have become crucial issues.

Here we report on a molecular epidemiological investigation of a *C. auris* cluster occurring in the same hospital where this yeast was first and only identified in Italy during 2019 [12]. The conjunction of WGS and patient trace data provided some notable insights into the genomic epidemiology of this fungal pathogen in our setting: (i) at present, its emergence in Italy is driven by one of the four major phylogeographically distinct lineages, namely clade I; (ii) the temporal analysis performed on sequenced genomes suggested that the introduction in our country was a relatively recent event (late May 2019); (iii) the genetic relatedness observed among most isolates was consistent with a nosocomial cluster sustained by a single clone, despite the interval of more than 6 months between less (August 2019) and more recent (February 2020) cases; (iv) clade-specific genetic signatures associated with high-level fluconazole resistance were identified; and (v) the detection of closely related clones, from patients admitted to different hospitals, with no epidemiological links, underscores that the dissemination of *C. auris* in our country could be broader than expected.

Over the past decade, *C. auris* has been detected across all major continents, and recent studies showed that in some European countries, like the UK and Germany [3,33], and in the US [27], where a near-nationwide spread has been observed, the emergence of this MDR yeast was contributed by isolates belonging to multiple genetic lineages (i.e., clade I, II, III). Conversely, our findings depicted a different epidemiological scenario in Italy, where a single lineage was recognized. Although WGS provided some insights into the phylogeny of characterized *C. auris* isolates, the introduction of this fungal pathogen in our country remains to be clarified, since the index patient had no history of recent travel or hospital admission abroad [12].

Both genomic and epidemiological data shed light on the putative transmission pathways in our setting, suggesting that unrecognized contaminated environments and/or colonized patients played a major role in creating long-term reservoirs and sustaining silent transmission chains, despite the bundle of infection-control interventions implemented at HSM after recognition of the index case (July 2019). Furthermore, genomic data suggested that one isolate (i.e., FG_GE03 from P3) was apparently not related with the cluster recognized at HSM, and likely represented an additional reservoir of ongoing transmission occurring in other regional facilities (e.g., Lavagna), thus posing major concerns for the control of this organism.

More importantly, the COVID-19 pandemic may have provided ideal conditions for the spread of *C. auris.* Indeed, in a context where the existing ICU capacity had been overwhelmed by the large number of COVID-19 patients requiring critical care and the conventional infection prevention and control measures were challenging (e.g., cohorting), the prolonged use of personal protective equipment (PPE) by the healthcare personnel may have inadvertently mediated a silent dissemination of this fungal pathogen. Epidemiological alerts of *C. auris* outbreaks occurring in healthcare facilities in the context of the COVID-19 pandemic have been recently documented also in Florida, Mexico, and India [34,35,36]. Of note, these reports consistently remarked the role that a possible low compliance to the guidelines for the correct use of PPE (e.g., experienced during anticipated or existing shortages) may have played in environmental contamination and transmission of *C. auris*, thus providing further evidence about major risk factors likely associated with an enhanced nosocomial spread of this organism during the pandemic. Furthermore, considering that critically ill COVID-19 patients tend to share risk factors (e.g., indwelling catheters) and underlying comorbidities with *C. auris* infections (e.g., diabetes mellitus, chronic kidney disease, intubation/mechanical ventilation, administration of broad-spectrum antibiotics, and systemic steroids), the spread of this fungal pathogen in the ICU setting raises major concerns [37]. Real-life data on the possible negative impact of the COVID-19 pandemic on antimicrobial stewardship programs has been recently reported [26]. In our hospital, following the increased number of cases observed in ward D, a systematic screening for *C. auris* colonization was started in mid-May 2020. Nevertheless, despite our attempts to monitor and control its spread, *C. auris* is proving difficult to eradicate and infection and/or colonization episodes continue to appear until now (November 2020).

All isolates characterized in this study showed a reduced susceptibility to voriconazole and were highly resistant to fluconazole, owing to the presence of missense mutations in the *ERG11*(K143R) and *TAC1B*(A640V) genetic loci, consistently with the recently reported association between high-level resistance to fluconazole and co-occurrence of these mutations [31]. Different *ERG11* hot-spot mutations (Y132F, K143R, and F126L or VF125AL) have been previously associated with different *C. auris* genetic lineages, with the *ERG11*(Y132F or K143R) being primarily found within the South Asian clade [15]. Interestingly, our findings underscored that a strong association between *ERG11*(K143R) and *TAC1B*(A640V) clearly subsists within the latter clade, corroborating the role of such mutations as lineage-specific resistance signatures. Besides azoles, all studied isolates showed a reduced susceptibility to amphotericin B, which represents a less common resistance trait in *C. auris* (Figure 2B) [38]. The variable susceptibility to amphotericin B is of particular concern and has been previously observed in outbreaks sustained by members of the South Asian clade [7].

## 5. Conclusions

Herein, we present the results of a molecular epidemiological investigation aimed at exploring the dynamics underlying the emergence and the dissemination of *C. auris* in a large teaching hospital in Italy. Present findings expand current knowledge on the population structure, genomic epidemiology, and resistance traits of this yeast. Its spread during the COVID-19 pandemic represents a worrisome phenomenon, and extreme caution is warranted for the management of critically ill COVID-19 patients developing infection or colonization by *C. auris.*

Continued efforts in identifying and in understanding the introduction and transmission dynamics of this emerging pathogen are critical to deliver timely and effective public health responses aimed at containing its spread within the healthcare system.

## Figures and Tables

**Table 1:** In vitro antifungal susceptibility profiles of characterized *C. auris* isolates (*n* = 10). The MIC range, MIC_50_, and MIC_90_ values (μg/mL) were reported.

Antifungal Drugs	MIC Range	MIC_50_	MIC_90_
Fluconazole	>256	>256	>256
Itraconazole	0.5	0.5	0.5
Voriconazole	4	4	4
Posaconazole	0.25	0.25	0.25
Caspofungin	0.06–0.25	0.25	0.25
Anidulafungin	0.12–0.25	0.25	0.25
Micafungin	0.06–0.12	0.12	0.12
Amphotericin B	2–4	2	4
Flucytosine	0.12–0.5	0.25	0.5

## Data Availability

Raw Illumina reads, complete and draft genomes of *C. auris* isolates sequenced in this study have been deposited in NCBI databases under BioProject no. PRJNA655187.

## Supplementary Materials

The following are available online at https://www.mdpi.com/2309-608X/7/2/140/s1, Table S1: Metadata of *C. auris* genome sequences included in this study.
