# Supplementary material for: Molecular Epidemiological Investigation of a Nosocomial Cluster of C. auris: Evidence of Recent Emergence in Italy and Ease of Transmission during the COVID-19 Pandemic
Source: J Fungi (Basel). 2021 Feb 15;7(2):140. doi: 10.3390/jof7020140 (PMC7919374; doi:10.3390/jof7020140)
Supplement: Supplementary file 1 [file jof-07-00140-s001.zip › Figure S1.docx]

Figure S1. Percent of Embryos Showing Sublethal Effects After Exposure to DCM- Extracted Pigments from Fungi at 24 and 120 hpf. Pigments showed different levels of toxicity under different test conditions, though generally longer time points and higher concentrations were related to higher toxicity levels. Zeros indicate there were no sublethal effects in that condition. Lack of sublethal deformation for S. cuboideum grown in maple media is due to death of all embryos at 24 hpf.
