# Supplementary material for: Molecular Epidemiological Investigation of a Nosocomial Cluster of C. auris: Evidence of Recent Emergence in Italy and Ease of Transmission during the COVID-19 Pandemic
Source: J Fungi (Basel). 2021 Feb 15;7(2):140. doi: 10.3390/jof7020140 (PMC7919374; doi:10.3390/jof7020140)
Supplement: Supplementary file 1 [file jof-07-00140-s001.zip › Figure S2.docx]

Figure S2. Percent of Embryos Showing Sublethal Effects After Exposure to Pigments from Fungi Grown in Liquid Media at 24 and 120 hpf. Zeros indicate there were no sublethal effects in that condition Lack of sublethal deformation information for *S. cuboideum* and *S. ganodermophthorum* in live and sterilized media, and *S. cuboideum* filtered media, is due to death of all embryos at 24 hpf. High levels of sublethal deformations were seen at 120 hpf for *Chlorociboria* species, whereas *Scytalidium* species were associated with such a high level of mortality that sublethal effects could not be measured under many conditions.
