# Supplementary material for: Molecular Epidemiological Investigation of a Nosocomial Cluster of C. auris: Evidence of Recent Emergence in Italy and Ease of Transmission during the COVID-19 Pandemic
Source: J Fungi (Basel). 2021 Feb 15;7(2):140. doi: 10.3390/jof7020140 (PMC7919374; doi:10.3390/jof7020140)
Supplement: Supplementary file 1 [file jof-07-00140-s001.zip › Supplemental figures.docx]

**Supplemental Figures**

Figure S1. Percent of Embryos Showing Sublethal Effects After Exposure to DCM- Extracted Pigments from Fungi at 24 and 120 hpf. Pigments showed different levels of toxicity under different test conditions, though generally longer time points and higher concentrations were related to higher toxicity levels. Zeros indicate there were no sublethal effects in that condition. Lack of sublethal deformation for *S. cuboideum* grown in maple media is due to death of all embryos at 24 hpf.

Figure S2. Percent of Embryos Showing Sublethal Effects After Exposure to Pigments from Fungi Grown in Liquid Media at 24 and 120 hpf. Zeros indicate there were no sublethal effects in that condition Lack of sublethal deformation information for *S. cuboideum* and *S. ganodermophthorum* in live and sterilized media, and *S. cuboideum* filtered media, is due to death of all embryos at 24 hpf. High levels of sublethal deformations were seen at 120 hpf for *Chlorociboria* species, whereas *Scytalidium* species were associated with such a high level of mortality that sublethal effects could not be measured under many conditions.
